# A Personal Health System for Self-Management of Congestive Heart Failure (HeartMan): Development, Technical Evaluation, and Proof-of-Concept Randomized Controlled Trial

**DOI:** 10.2196/24501

**Published:** 2021-03-05

**Authors:** Mitja Luštrek, Marko Bohanec, Carlos Cavero Barca, Maria Costanza Ciancarelli, Els Clays, Amos Adeyemo Dawodu, Jan Derboven, Delphine De Smedt, Erik Dovgan, Jure Lampe, Flavia Marino, Miha Mlakar, Giovanni Pioggia, Paolo Emilio Puddu, Juan Mario Rodríguez, Michele Schiariti, Gašper Slapničar, Karin Slegers, Gennaro Tartarisco, Jakob Valič, Aljoša Vodopija

**Affiliations:** 1 Department of Intelligent Systems Jožef Stefan Institute Ljubljana Slovenia; 2 Department of Knowledge Technologies Jožef Stefan Institute Ljubljana Slovenia; 3 Health Unit Atos Research and Innovation (ARI) Atos Spain S.A. Madrid Spain; 4 Department of Cardiovascular, Respiratory, Nephrological, Anesthesiological and Geriatric Sciences Sapienza University of Rome Rome Italy; 5 Department of Public Health and Primary Care Ghent University Ghent Belgium; 6 Meaningful Interactions Lab KU Leuven Leuven Belgium; 7 SenLab d.o.o. Ljubljana Slovenia; 8 Institute for Biomedical Research and Innovation National Research Council of Italy Messina Italy; 9 Department of Communication & Cognition Tilburg School of Humanities and Digital Sciences Tilburg University Tilburg Netherlands

**Keywords:** congestive heart failure, personal health system, mobile application, mobile phone, wearable electronic devices, decision support techniques, psychological support, human centered design

## Abstract

**Background:**

Congestive heart failure (CHF) is a disease that requires complex management involving multiple medications, exercise, and lifestyle changes. It mainly affects older patients with depression and anxiety, who commonly find management difficult. Existing mobile apps supporting the self-management of CHF have limited features and are inadequately validated.

**Objective:**

The HeartMan project aims to develop a personal health system that would comprehensively address CHF self-management by using sensing devices and artificial intelligence methods. This paper presents the design of the system and reports on the accuracy of its patient-monitoring methods, overall effectiveness, and patient perceptions.

**Methods:**

A mobile app was developed as the core of the HeartMan system, and the app was connected to a custom wristband and cloud services. The system features machine learning methods for patient monitoring: continuous blood pressure (BP) estimation, physical activity monitoring, and psychological profile recognition. These methods feed a decision support system that provides recommendations on physical health and psychological support. The system was designed using a human-centered methodology involving the patients throughout development. It was evaluated in a proof-of-concept trial with 56 patients.

**Results:**

Fairly high accuracy of the patient-monitoring methods was observed. The mean absolute error of BP estimation was 9.0 mm Hg for systolic BP and 7.0 mm Hg for diastolic BP. The accuracy of psychological profile detection was 88.6%. The F-measure for physical activity recognition was 71%. The proof-of-concept clinical trial in 56 patients showed that the HeartMan system significantly improved self-care behavior (*P*=.02), whereas depression and anxiety rates were significantly reduced (*P*<.001), as were perceived sexual problems (*P*=.01). According to the Unified Theory of Acceptance and Use of Technology questionnaire, a positive attitude toward HeartMan was seen among end users, resulting in increased awareness, self-monitoring, and empowerment.

**Conclusions:**

The HeartMan project combined a range of advanced technologies with human-centered design to develop a complex system that was shown to help patients with CHF. More psychological than physical benefits were observed.

**Trial Registration:**

ClinicalTrials.gov NCT03497871; https://clinicaltrials.gov/ct2/history/NCT03497871.

**International Registered Report Identifier (IRRID):**

RR2-10.1186/s12872-018-0921-2

## Introduction

### Background and Motivation

Congestive heart failure (CHF) is a disease in which the heart cannot pump enough blood to supply oxygen and nutrients to the body. The main symptoms are shortness of breath (dyspnea), diminished ability to exercise, fatigue, and swelling in the feet and legs (edema). The lifetime risk of developing CHF ranges from 20% to 33%, and only approximately half of patients survive for more than 5 years after diagnosis [1]. As CHF is frequently the end stage of various conditions that affect left ventricular function and cannot be cured, the focus of the treatment is to prevent deterioration, manage symptoms, and maintain a good quality of life [2].

The management of CHF includes multiple medications, appropriate exercise, diet (paying particular attention to fluids and salt), management of body weight, and abstaining from alcohol and smoking. As the average age at CHF diagnosis is 74 (SD 14) years [3], 25% to 80% of the patients are affected by cognitive impairment [4], a third of them have depression or anxiety [5], and other comorbidities are also common, they often find it difficult to manage the disease on their own [6]. Cardiac rehabilitation programs are either not available or poorly attended—participation in Europe is approximately 20% [7]. Therefore, the relevant alternatives are technological solutions to support the management of CHF.

Approximately 64 million people live with CHF globally [1], and the economic burden of their disease amounts to more than 100 billion US $ annually [8]. This is a strong incentive to improve CHF management. In addition to medications, implantable devices (mainly pacemakers and defibrillators) are already established treatment options [9]. Another option is telemonitoring, but its benefits in CHF are uncertain [9]. Another option is mobile health (mHealth) solutions, whose benefits in CHF are poorly explored (see the Related Work section) but have a strong backing of the market: the mHealth market in 2019 was US $46 billion and grew by 22% annually [10] (compared with the telemonitoring market of US $2 billion with 13% growth [11] and the more mature implantable devices market of US $23 billion and 8% growth [12]).

In the HeartMan project, we developed a comprehensive personal health system for the self-management of physical and psychological aspects of CHF. The first step was to analyze evidence-based medical requirements and—following the human-centered design process—to elicit requirements related to everyday management of CHF from the patients themselves. We then developed a mobile app comprising a decision support system (DSS) and several intelligent data analysis modules. A web application for medical professionals has also been developed. Finally, the system was evaluated in a proof-of-concept trial that assessed both its effectiveness and patient perception.

### Related Work

In 2018, a systematic review was devoted to mobile apps supporting the self-management of CHF [13]. The authors surveyed 10 leading paper repositories for papers on interventions that used a mobile platform, evaluated them with a randomized controlled trial or a similar design, and provided usability or efficacy results. Papers on telecare and structured telephone support were excluded. In total, 18 papers meeting the inclusion and exclusion criteria were included in the review. The authors also searched Google Play and Apple App Store for health care apps by including “heart failure” as a keyword. After excluding apps that track only blood pressure (BP) and/or heart rate, a total of 26 apps were downloaded and evaluated with respect to the quality of self-management components included in the apps and quality of the user experience provided by the apps.

According to the authors of the review [13], most apps are poorly designed and do not include all the necessary components for the self-management of CHF. Indeed, only 2 apps—Heart Failure Storylines [14] and HeartMapp [15]—include exercise interventions, which is one of the most important aspects of CHF management. The Heart Failure Storylines app is perhaps the most complete one that can be currently found in the market. It provides medication reminders, a symptom tracker, keeps a record of vital signs, and tracks physical activity and daily moods. Nevertheless, the interventions provided by the app are poorly personalized (except for medication reminders) because the app does not consider the patients’ psychophysical state, making the usefulness of such interventions questionable [9,16]. The HeartMapp app provides personalized interventions, but it is quite basic and is not adapted to the patients’ psychophysical state. The app was tested in a randomized controlled trial with only 18 participants (intervention group, n=9) [17].

We searched the Google Play and Apple App Store for apps that were not included in the review. We found 6 apps that were published after the review. Five of these apps include only educational materials [18-23], whereas 1 app provides only guidance on medication therapy [24]. In short, no new apps provide a comprehensive solution for CHF management.

## Methods

### Collection of Requirements and Human-Centered Design

#### Medical Requirements

The first step in designing the HeartMan system was to study the state-of-the-art medical knowledge on CHF self-management. A systematic review of the available literature was performed to identify parameters that predict the hard outcomes of mortality and hospitalization in patients with CHF as well as variables that affect the patient-reported outcome of quality of life in this patient group [25]. We further selected those parameters that are modifiable by self-care behaviors that the HeartMan system can recommend. These modifiable parameters are primarily clinical parameters (eg, body mass index, BP, heart rate), physical capacity, medication use, characteristics of CHF (eg, fluid retention), and mental health (eg, depression, anxiety). We then screened relevant medical guidelines for CHF, focusing on nonpharmacological recommendations and lifestyle advice, to identify the best approaches for modifying these parameters [26] and incorporated these into the HeartMan DSS. We designed an exercise training and nutrition program (including diet and fluid intake restrictions) to influence physical capacity, clinical parameters, and fluid retention. Medication adherence is expected to be enhanced through DSS, providing reminders, disease education, and self-monitoring. Finally, cognitive behavioral therapy and mindfulness exercises were included to improve mental health and self-management. Management guidelines for comorbidities were also taken into account, as many patients with CHF have conditions such as diabetes, atrial fibrillation, and chronic obstructive pulmonary disease.

An additional source for developing the medical requirements was data from the Chiron project [27], a previous telemonitoring study in patients with CHF focusing on short-term outcomes of subjective well-being on a daily basis. Data mining analysis suggested environmental parameters, that is, ambient conditions such as temperature and humidity, to play a role in predicting day-to-day changes in perceived health. This was incorporated into an additional module of DSS.

#### User Requirements

As our goal was not only to provide medically relevant advice but also to design the HeartMan system to be useful and well accepted by the patients, we adopted a human-centered design [28]. This approach involves users throughout the design process, focusing on their perspective and needs. In our case, it consisted of a thorough analysis of patients’ context of use, which took place in three stages in Belgium and Italy. The first stage was a diary study, in which patients kept a diary for a period of 10-14 days (n=19 in Belgium; n=18 in Italy). The diary contained questions and assignments related to everyday activities and habits, such as patients’ experience, disease management, and their social network. The second stage was a follow-up interview study conducted with most patients who participated in the diary study (n=14 in Belgium; n=15 in Italy). In this interview study, patients participated in semistructured interviews in which the output of the diary study was discussed in detail. This analysis resulted in a rich, qualitative description of patient characteristics as well as the patient experience regarding disease management, the challenges related to therapy adherence, lifestyle changes as a result of being a CHF patient, and relationships with caregivers. These insights were translated into concrete user requirements for the HeartMan system, which served, together with the medical requirements, as the starting point for the third stage: the design and evaluation of a series of prototypes with both patients and caregivers. In this process, several design trade-offs were made regarding patient autonomy, technology appropriation, and patient well-being [29]. The main patient characteristics that were found to impact the design of the HeartMan system were the patient’s digital literacy, perception of empowerment, and existing therapy adherence habits.

For medical professionals, a web portal was developed, allowing them to follow up on the patients’ data gathered by the HeartMan system. This prototype was developed and evaluated using a separate human-centered design process. In this process, various stakeholders (including cardiologists, nurses, dieticians, psychologists, and physiotherapists) offered insights into the needs and requirements related to the follow-up of patients with CHF based on the HeartMan monitoring data.

### System Overview

**Figure 1:**
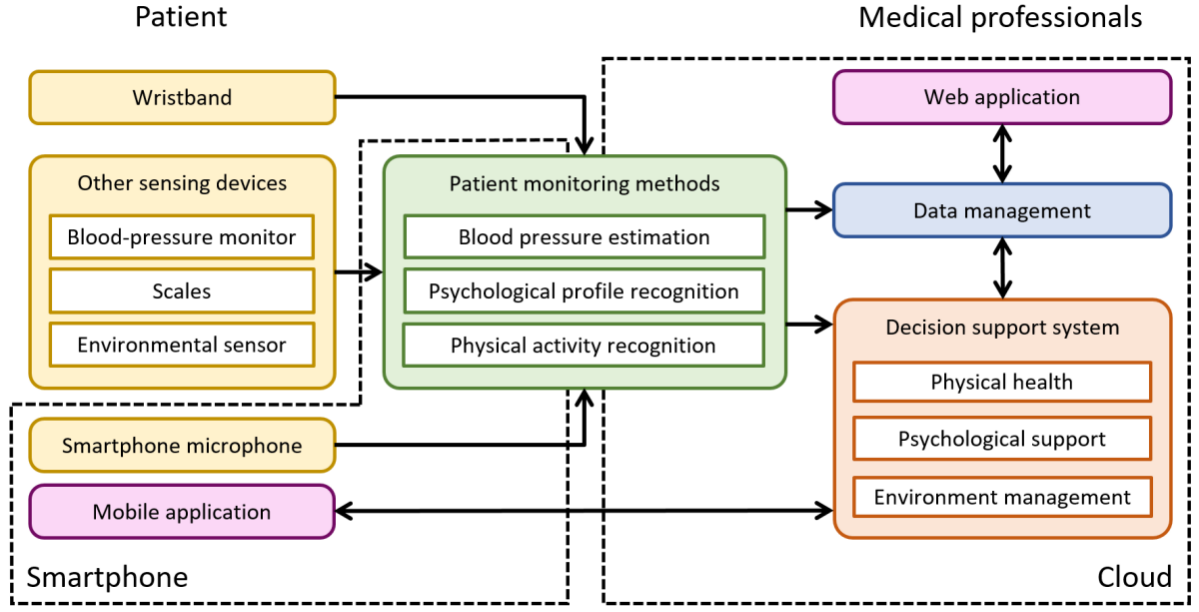
The logical architecture of the HeartMan system.

In the HeartMan system designed as described in the previous section, sensing devices collect information about the patient, patient monitoring methods further interpret some of this information, and a DSS recommends actions based on the (interpreted) information. The recommendations are presented to the patient via a mobile app, and medical professionals have access to the system via a web application. A diagram presenting an overview of the system is presented in Figure 1.

The sensing devices (yellow in Figure 1) are custom sensing wristbands, off-the-shelf BP monitors, weight scales, and environmental sensors that measure temperature and humidity. According to the medical requirements, heart rate (obtained from the photoplethysmogram [PPG] signal), BP, weight, and ambient temperature and humidity are important determinants of the health and well-being of patients with CHF. As it would be relevant to monitor BP more frequently than once per day (which can be expected with a regular BP monitor), we developed a method to estimate BP continuously from the PPG signal (green). Owing to the importance of psychological support for patients with CHF, we also developed a method to recognize their psychological profile from the heart rate, heart-rate variability, and voice recorded with the smartphone. Finally, the accelerometer in the wristband is used to recognize the patient’s physical activities, which allows the initiation of psychological interventions at the appropriate moment. As the accelerometer provides the greatest volume of data of all the sensors, this last method is implemented on the smartphone, whereas the previous 2 reside in the cloud.

All patient information is fed into the DSS and stored in the cloud (blue in Figure 1). The DSS has three components, the first of which is an expert system that helps patients manage their physical health (exercise, nutrition, medications, and self-monitoring). The second is another expert system that provides psychological support (elements of cognitive behavioral therapy and mindfulness). The third uses predictive models (based on the previously mentioned Chiron data) to recommend actions related to temperature and humidity that are expected to improve patients’ well-being. The first 2 components rely on expert knowledge because it is well established how the aspects of the CHF management they address should be tackled. The last one relies on data and predictive modeling because we had relevant data available, but there is little expert knowledge on the effect of the environment on the well-being of patients with CHF.

The recommendations provided by the DSS are shown in the mobile app (purple in Figure 1), which also collects inputs from the patients. Medical professionals can use a web application to view information collected from sensing devices as well as the patients’ adherence to recommendations. Although the content of recommendations is mostly based on the medical requirements, the way information is presented via the 2 applications was heavily influenced by the users’ inputs obtained during the human-centered design process.

### Patient-Monitoring Methods

#### The HeartMan Wristband

The wristband used by the system includes a PPG sensor, which provides information on the heart rate and beat-to-beat intervals in addition to the raw data, tri-axial accelerometer, and temperature sensor. It communicates with the HeartMan app via Bluetooth Low Energy 4.1. Its battery life is sufficient for a full day of operation, while continuously streaming sensor data to the phone. It features a liquid crystal display and vibration motor, which can be used to deliver urgent notifications to the user, such as about too high or low heart rate during exercise.

#### BP Estimation

Continuous BP estimation is well researched when 2 signals, typically ECG and PPG, are available, as the pulse transit time between 2 points on the body is highly correlated with the BP [30,31]. In HeartMan, we aimed to use a single wristband PPG sensor [32,33], as this is the most convenient for the patients. However, such a sensor typically has a modest sampling frequency, the sensor-to-skin contact is often compromised due to movement, and the wrist area exhibits less pulsatility compared with a fingertip, making this approach challenging.

To obtain high-quality parts of the PPG waveform, the signal was preprocessed. The first step was zero-mean unit-variance normalization. Outlier samples above 3 SDs from the local median (10-sample window) were removed using a Hampel filter. Afterward, the signal was filtered using a fourth-order Butterworth band-pass (0.5-4.0 Hz) filter. Then, a transformation based on the first-order derivative was used to detect systolic peaks and diastolic valleys in between. Once the valleys were detected, the signal was traversed with a sliding window, and a template was created as the average of all cycles in a window. Following this, each individual cycle was compared with the template using several metrics. This allowed for the detection of segments where the signal was stable with only a few artifacts, while also allowing for individual bad cycles in an otherwise good segment to be discarded [34].

After preprocessing, per-cycle temporal features describing the cycle shape were computed based on related work [35] and further expanded with some features from the frequency domain. The latter were computed from a window centered on a cycle and extending 5 seconds before the cycle start point and 5 seconds after the end point. Most of the temporal morphologic features rely on high-quality waveform, exhibiting a clear systolic and diastolic peak, as they were designed for fingertip PPG devices in a controlled setting. The HeartMan wristband signal is generally of lower quality, so we focused on frequency domain features, which are more robust, as they are computed from longer windows and not on a per-cycle basis. In addition, as some morphological features were infeasible to compute from the HeartMan wristband data, we additionally leveraged information from the accelerometer, which tells us about the person’s physical activity. We considered some commonly used features computed from the three-axis accelerometer, which are known to work well in separating a person’s activities [36]. We decided on this because having information about a person’s activity might prove useful for BP estimation, as the cardiovascular response of the body changes during intense physical activity compared with the state. This fact differentiates this work from previous work dealing with similar problems, as related work often focuses on PPG signals without considering the person’s activity, which can be reflected in the accelerometer signal [37]. Finally, heart rate was also used as a feature to inform us about a person’s cardiac activity. All these features were fed into regression models that estimated systolic BP (SBP) and diastolic BP (DBP). Several algorithms implemented in the Scikit-learn toolbox [38] were used to train the models, some of which are compared in the Results section.

#### Psychological Profile Recognition

The development of technological interventions for behavior changes as well as growing interest in affective computing have resulted in various attempts to recognize psychological states from sensor data. Some authors [39] used mobile phones to analyze user voices and classify their emotions (happy, sad, fear, anger, and neutral). Others have focused on stress, dementia, and cognitive dysfunctions, relying more on wearable devices that sense the heart rate, electrodermal activity, skin temperature, and acceleration [40,41].

The HeartMan system combines the patient’s voice obtained during a structured weekly phone interview with an informal caregiver with heart rate features, which can be obtained from the HeartMan wristband. The speech data were preprocessed to normalize the different acoustic properties, such as higher volume and background noise, using standard techniques [42]. The features extracted from the speech are the fundamental frequency (pitch), mel-frequency cepstral coefficients, and the smoothed energy. The mean, SD, range, maximum, and minimum were computed for each base speech feature. In addition, the heart rate and heart rate variability represented by the root mean square of successive differences between heartbeats were extracted. The features are then fed into a machine learning model that recognizes motivated, anxious, and depressed psychological profiles. All the data were preprocessed and analyzed using MATLAB and R software.

#### Physical Activity Recognition

Physical activity recognition is a relatively mature field, although the requirements of HeartMan present some challenges. As the purpose was to initiate psychological interventions, it was most relevant to recognize eating and to distinguish resting from walking and more intense activities. Eating recognition is quite difficult and rarely addressed in the literature, whereas wrist—being able to move independently from the body—is not the best location for recognizing the intensity of activity.

Similar to the previous 2 patient-monitoring methods, this method also uses machine learning. The stream of acceleration data is first low-pass filtered to remove noise and then band-pass filtered to remove the gravitational component, retaining the component due to dynamic human motion. The stream was then segmented into 2-second windows. In each window, the low-pass filtered data are used to compute features related to the orientation of the sensor, whereas the band-pass filtered data are used to compute the features related to the motion of the sensor. A total of 90 features were extracted [37]. Some describe the intensity and shape of the acceleration signal, such as the mean, variance, skewness, and kurtosis. Others have a physics-based interpretation, such as changes in velocity and kinetic energy. The rest are based on expert knowledge, such as the number of peaks in the signal and the number of times the signal crosses its mean value. The features are fed into a machine learning model that returns one of the following activities: rest, standing, walking, Nordic walking, running, other exercise, eating, washing hands or face, household chores (whole-body movement), and light hand activities (hand movement). The model was built using the random forest algorithm implemented in the Weka toolkit [43].

### DSS

#### Expert System for Physical Health Management

##### Exercise

The HeartMan DSS administers a comprehensive exercise program [44] according to the established medical guidelines [16]. Before starting the exercise program, the patients were expected to perform a cardiopulmonary exercise (cycloergometry) or a 6-min walking test to assess their physical capacity. On this basis, the physical capacity of each patient is assessed as *low* or *normal*, which affects the exercise planning.

###### Weekly Exercise Planning

The DSS proposes a weekly exercise plan for each patient, consisting of endurance and resistance exercises. The DSS suggests the frequency (times per week), intensity, and duration of each exercise type. The suggestions are based on the patient’s physical capacity, the number of active weeks in the program, and the current frequency and intensity. They are also based on the patient’s psychological profile: the difficulty increases more gradually for depressed patients, which is in line with the *shaping* technique suitable for this profile. For instance, low-capacity patients start with very light 10- to 15-min endurance exercises twice per week. According to the patient’s progress, these parameters may change with time, typically by increasing the frequency and intensity of exercises, if the patient agrees. The planning process is governed by an expert system that consists of 2 rule-based models, developed using a qualitative multicriteria method decision expert [45] and described in more detail in our earlier work [44].

###### Exercise Sessions

**Figure 2:**
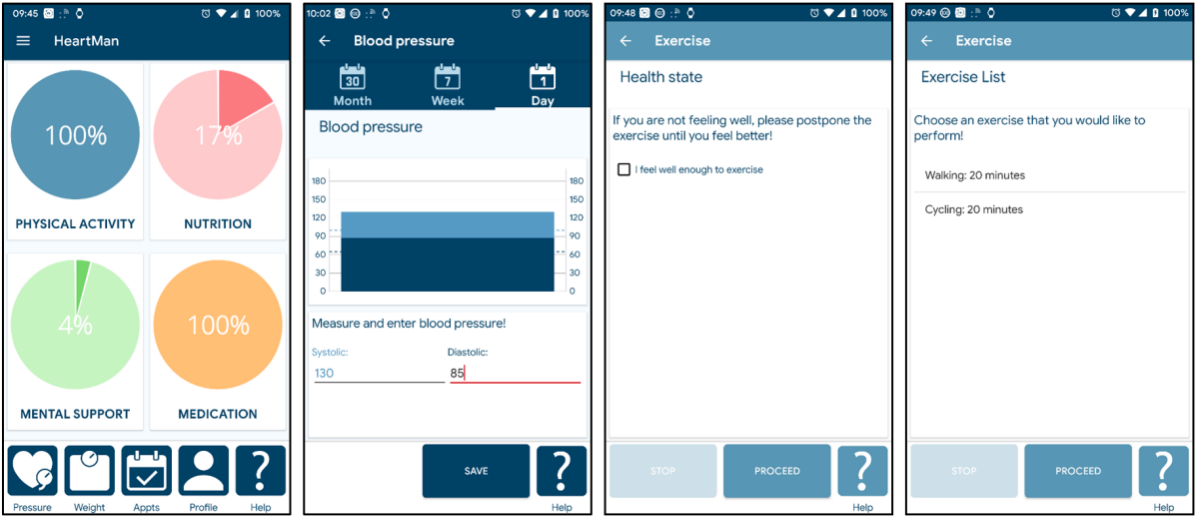
Exercise-related screens of the HeartMan app: the main screen, blood pressure input before the exercise, health check before the exercise, and exercise list.

Before the start of each exercise session, the HeartMan DSS checks whether the patient’s BP and heart rate are in a safe range and whether the patient feels well enough to exercise. If the exercise is allowed, a list of exercises is shown to the patient, who can then select the preferred exercise. This is illustrated in Figure 2. Typical endurance exercises involve walking and cycling, whereas resistance exercises aim to strengthen the patient’s arms, legs, and body. After selecting the exercise, a detailed description (text or graphical) was provided. During the exercise, the heart rate and SBP were continuously measured using the wristband. Patients are advised to stop the exercise in cases of symptoms or measurements outside a safe range. During endurance exercises, the DSS uses the wristband display to suggest an increase or decrease in pace based on the heart rate. After completing the exercise, the patients can rate their feeling of intensity, which is used in the weekly planning to decide whether to increase the intensity.

##### Nutrition

To provide appropriate nutrition advice, the DSS requires the following medical information: the patient’s BMI, whether the patient has diabetes, and the prescribed amount of liquid intake. Next, the DSS creates a personalized questionnaire to be answered by the patient; it includes general questions about healthy nutrition and specific questions about the patient’s eating and drinking behavior. On this basis, the DSS assesses the level to which topics (about breakfast, lunch, dinner, fat and cholesterol, fluid intake, salt, diabetes, and medication) are understood by the patient. Finally, the patient received feedback in terms of positive reinforcement messages (for well-understood topics), educational statements (for misunderstood general topics), and advice on how to modify the diet to make it healthier (for misunderstood eating behavior topics).

##### Self-Monitoring and Medication

Patients with CHF are required to measure their BP, heart rate, and daily weight. The HeartMan system reminds them of this and warns if the measurements are outside the safe ranges. It also reminds the patients to take their medications and helps them fill the weekly pillbox (if they use one). It periodically asks the patient about the number of pills remaining in the pillbox and assesses medication adherence based on the deviation from the expected number.

#### Expert System for Psychological Support

In most cases, CHF diagnosis requires substantial changes in daily life and habits, such as dietary modifications and increased physical activity. Combined with psychological distress, which also often follows the diagnosis, patients can face an intrusion of distorted beliefs and negative automated thoughts that cause them to feel unable to pursue a goal [46]. Sometimes a vicious circle called cognitive dissonance is triggered: a conflict between their desire to be healthy on one hand and practicing unhealthy behaviors for short-term comfort on the other hand. In the long run, this results in poor adherence to self-management guidelines as well as psychological discomfort [47].

The psychological DSS is designed to select the appropriate strategy to improve patients’ psychological well-being and adherence to physical exercise and dietary guidelines. The strategy is adapted to the user’s psychological profile, as discussed in the section on psychological profile recognition. The DSS provides cognitive behavioral interventions and mindfulness exercises that are modified according to a weekly plan. These exercises are suggested daily, at a time when the user engaged in a physical activity expected to make them receptive to the suggestion. The relevant activities are eating, walking, and sitting, as discussed in the Physical Activity Recognition section.

##### Cognitive Behavioral Therapy

This is a combination of behavioral and cognitive techniques developed to reduce anxiety and depressive symptoms, which tend to make patients less motivated, tired, and less energetic. The DSS provides specially designed messages intended to align the patients’ actions with their desires, as shown in the examples in Table 1. These messages are formulated according to the principles by Festinger [48] of *cognitive consequences of forced compliance* for the motivated profile, *free choice* for the anxious profile, and *effort justification* for the depressed profile.

**Table 1:** Examples of cognitive behavioral therapy messages about physical exercises for three different psychological profiles.

Psychological profile	Festinger principle	Example message
Motivated profile	Cognitive consequences of forced compliance	I should perform physical exercise to obtain benefits similar to those from medications
Anxious profile	Free choice	Walking for 10 min and watching TV^a^ are two ways to relax. Walking improves your heart health, whereas TV does not
Depressed profile	Effort justification	Walking for 10 min will bring benefits similar to those obtained from medication

^a^TV: television.

##### Mindfulness

**Figure 3:**
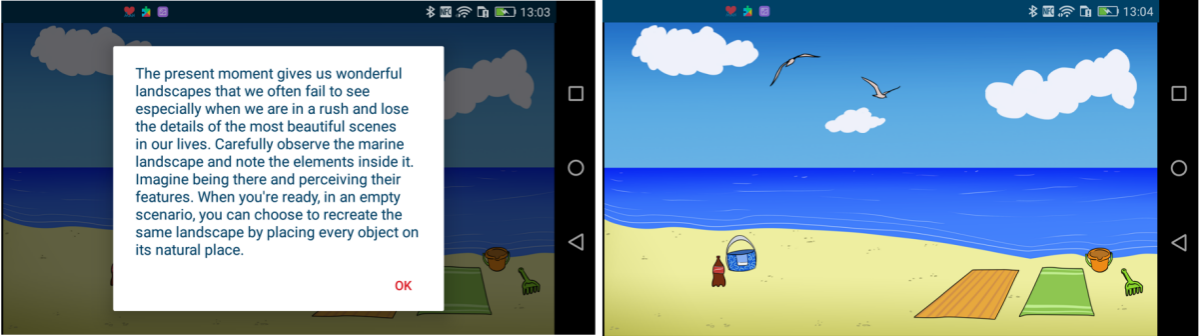
Mindful game "World Sense".

Mindfulness exercises enhance patients’ awareness of their present condition and help them disassociate (unhealthy) emotional and behavioral responses from physical sensations and thoughts. Mindfulness exercises consisted of the following:

- Games to deal with intrusive thoughts (eg, loss of independence, feeling restricted in daily activities), as shown in Figure 3.
- Audio recordings dealing with the perception of the patient’s body and breathing exercises.
- Mindful messages that help the patient focus on a mindful moment. These messages are contextualized as follows: mindful walking when the user is walking, mindful breathing when the user is sitting, mindful eating when the user is eating, and mindful listening and observing when the user is either walking or sitting.

#### Predictive Models for Environment Management

Unlike the DSS approaches used for physical health management and mental support, which mainly rely on expert knowledge, a data-based approach was developed for environment management. We used data from the Chiron project [27], which consists of features describing the patient’s situation and their self-reported feeling of health. The features are physiological (eg, heart rate, BP) and environmental (eg, temperature, humidity) and very similar to those available to the HeartMan system.

In the first step, we built a machine learning model that could predict the feeling of health from the features. We used the random forest algorithm implemented in the Weka toolkit [43]. The accuracy of distinguishing between good and bad feelings of health was 83.2%. We also divided the features into modifiable, correlated (with modifiable), and uncorrelated. We build linear regression models that can predict each correlated feature from the modifiable ones.

In the second step, we set up a multi-objective optimization problem, where we searched for minimal modifications of modifiable features that change the feeling of health from bad to good. For each solution, the correlated features were predicted using linear models, and the admissibility of the solution was checked using the feeling-of-health model. The objectives were the sum of the volumes of modifications needed and the number of modified features, as making smaller modifications to a smaller number of features is easier. To solve this problem, we used the multi-objective evolutionary algorithm Nondominated Sorting Genetic Algorithm-II [49].

For more than half of the cases, we were able to find a solution where changing only 1 or sometimes 2 modifiable features would improve the patient’s feeling of health. For some cases, we needed to change more features, and for a minority of the cases, no suitable modification could be found. More detailed results can be found in our previous study [50].

### Implementation

**Figure 4:**
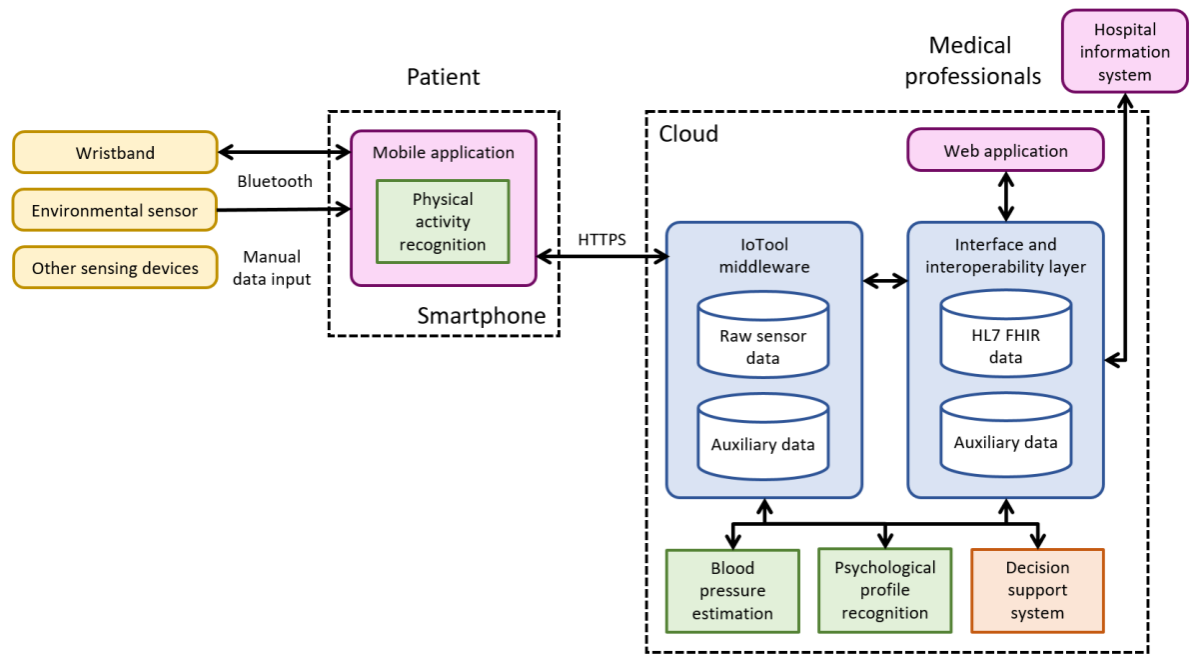
The physical architecture of the HeartMan system.

All the patient-monitoring and decision support modules were integrated into the HeartMan system together with apps for patients and medical professionals. The architecture of the integrated system is illustrated in Figure 4. The wristband and environmental sensor are connected to the mobile app via Bluetooth Low Energy. The mobile app, which includes the physical activity recognition, runs on the smartphone. Physical activity recognition was placed there because it was more efficient to do so than to transmit all the raw accelerometer data to the cloud. On the right side are cloud services, which include BP estimation, psychological profile recognition, and DSS. These were placed entirely in the cloud because they required less raw sensor data, and implementation was easier. Cloud services were installed inside the hospital to comply with the general data protection regulation.

The data from the mobile app were received by the IoTool middleware [51], whose main purpose was the retrieval of sensor data from smartphones and connected devices, and its storage in a database in the cloud. As it can send data in both directions, it was also used to synchronize application data (such as exercise plans, patient inputs, and push notifications) between the smartphone and the cloud. In this way, the app received the information needed to support each patient on a weekly basis and was then largely independent from the internet for a week. Finally, IoTool can apply arbitrary transformations to sensor data, creating so-called virtual sensors: this capability was used for physical activity recognition, which was implemented as an IoTool virtual sensor transforming acceleration data into physical activities.

Most raw sensor data were retained in the IoTool database for offline analysis, whereas the data required for HeartMan operation were passed through the interface and interoperability layer, stored using the HL7 FHIR (fast health care interoperability resources) standard for health data exchange [52] if applicable and made available to other services: BP estimation, psychological profile detection, and the DSS. Each of these services reads inputs from and writes outputs to the central storage via the interface and interoperability layer. The data that needed to be sent back to the smartphone were stored in the IoTool database for synchronization. The interface and interoperability layer also provided data to the web application for medical professionals and enabled interoperability with hospital information systems. To do so, it complied with the FHIR REST (representational state transfer) API (application programming interface) specification [52].

**Figure 5:**
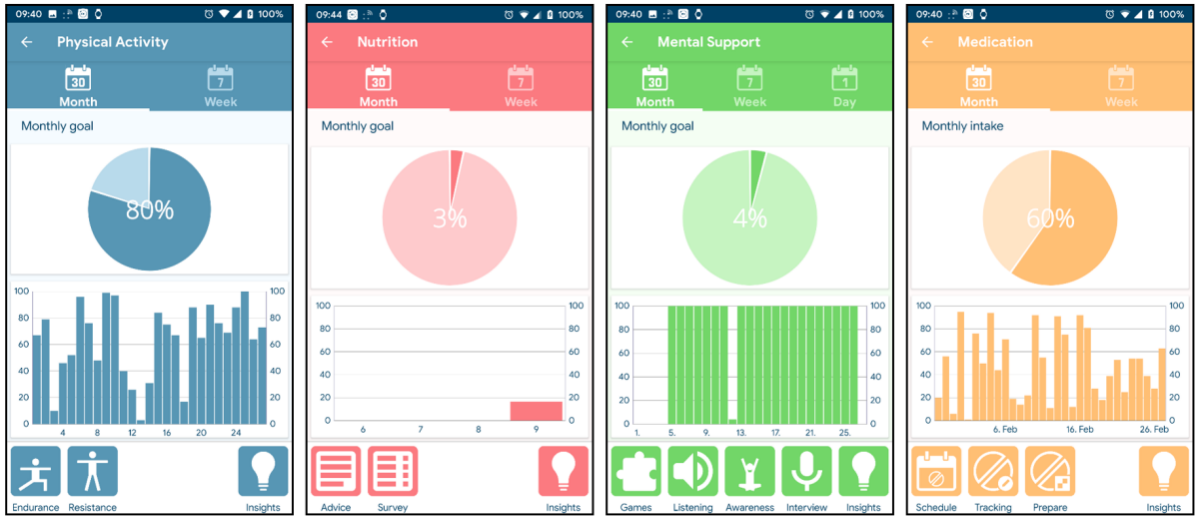
Dashboards of the HeartMan mobile app for physical activity, nutrition, mental support, and medication management.

The HeartMan mobile app is divided into four sections according to the main topics identified in the medical and user requirements. The respective dashboards are shown in Figure 5. They prominently show the percentage of monthly or weekly activities already performed, which corresponds to the adherence to the HeartMan-suggested self-management at the end of the month or week. The buttons at the bottom trigger various activities, and there is also an Insights section that provides general education on CHF.

**Figure 6:**
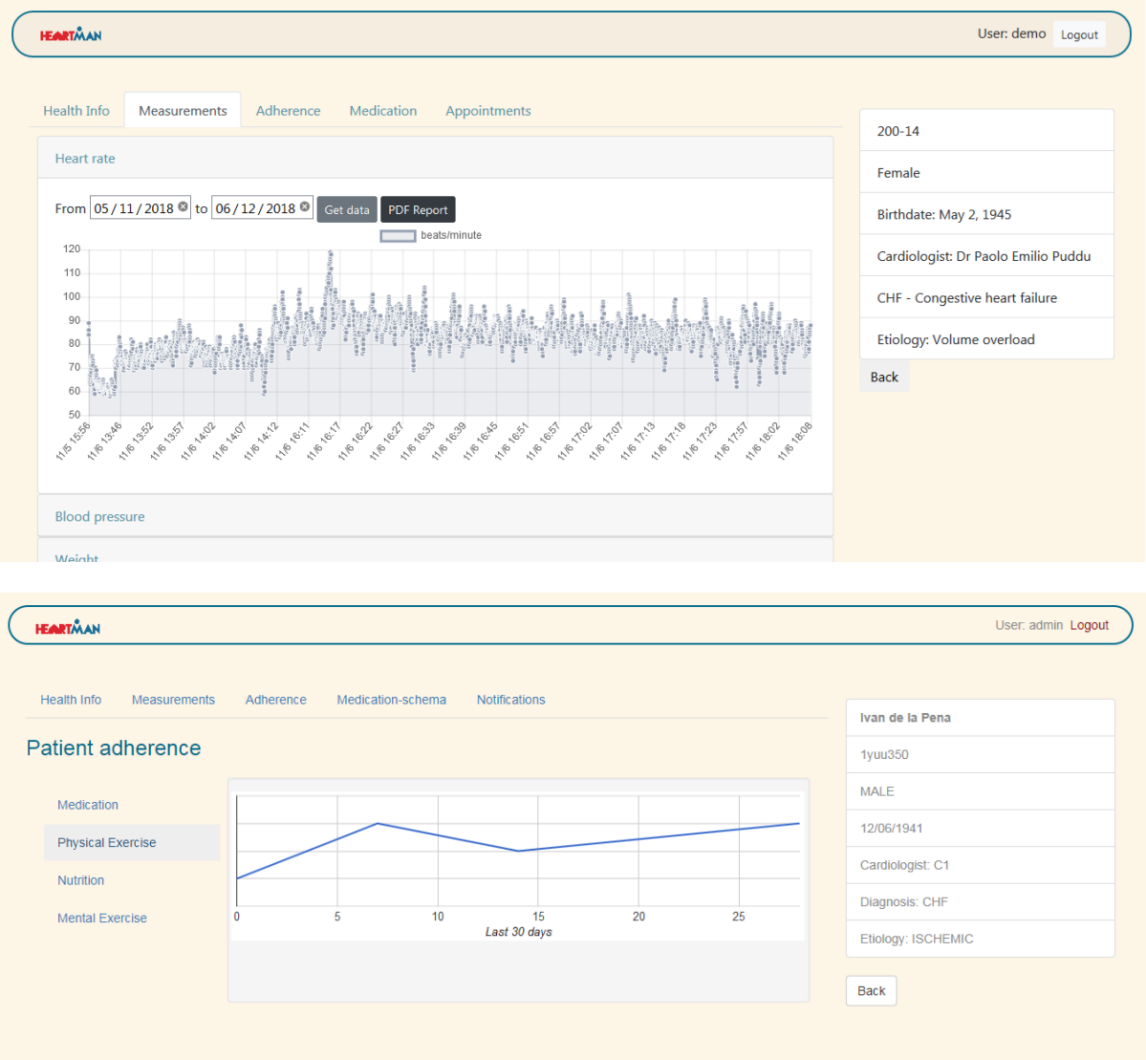
Screenshots of the HeartMan web application for medical professionals: heart rate measurements (upper) and adherence to the HeartMan-suggested self-management (lower).

The web application for medical professionals shows the patients’ clinical information, measurements of heart rate, BP, and weight, and their adherence to the HeartMan-suggested self-management. It also enables the management of medications, with the updated medication plan displayed in the mobile app. Screenshots of the web application are shown in Figure 6.

## Results

### Accuracy of the Patient-Monitoring Methods

#### BP Estimation

For the first BP estimation test, we collected a data set from 22 healthy subjects (ages 22 to 39 years, 6 women and 16 men) using the Empatica E4 wristband [53]. They wore the wristbands continuously throughout the day and were told to measure their ground truth BP with a certified Omron device every 30 minutes. Each ground truth BP value was attributed to the PPG signal 30 seconds before and after each measurement was made. Leave-one-subject-out evaluation was conducted, and the mean absolute error (MAE) between the estimated and ground truth SBP and DBP was used as the evaluation metric. Several regression algorithms were compared against a baseline dummy regression model, which always outputs the average SBP and DBP of the training set.

**Figure 7:**
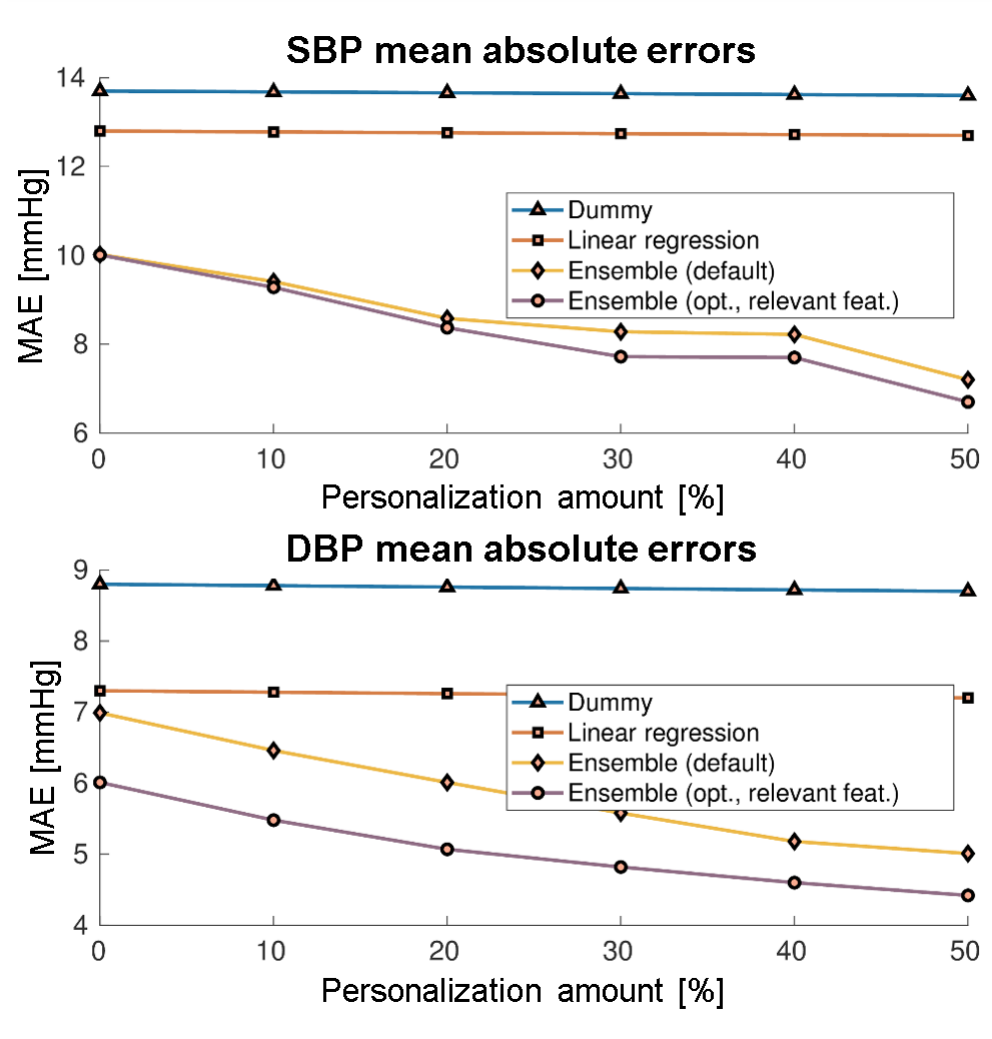
Mean absolute error of systolic blood pressure and diastolic blood pressure estimation in the leave-one-subject-out experiment using the Empatica E4 wristband. DBP: diastolic blood pressure; MAE: mean absolute error; SBP: systolic blood pressure.

Using the Empatica E4 data, the initial errors of ensembles of regression trees were approximately 10 mm Hg for SBP and 6 mm Hg for DBP, as shown in Figure 7. The results were further improved using personalization, achieving errors of 6.70 mm Hg for SBP and 4.42 mm Hg for DBP, suggesting that the connection between PPG and BP is person-specific and that a general model is difficult to derive.

As the HeartMan wristband was a prototype intended for wide use by patients (as opposed to the Empatica E4, which is a high-cost research device), the quality of the PPG signal was lower. Therefore, we built person-specific models using the data collected from the HeartMan trials. The patients wore the wristband and were instructed to measure their BP daily with a certified device, so we matched the PPG and BP data as in the previous experiment. We used a train-test split of 70% to 30% to ensure no data leakage. We compared a number of regression algorithms with random forest performing the best, as shown in Table 2.

**Table 2:** MAEs of systolic blood pressure and diastolic blood pressure estimation of personalized models from the HeartMan trial.

Algorithm	MAE^a^ of systolic blood pressure (mm Hg)	MAE of diastolic blood pressure (mm Hg)
Baseline dummy (mean)	11.4	8.9
Decision tree	13.1	10.1
k-nearest neighbors	10.6	7.5
Support vector regression	11.3	8.5
Random forest	9.0	7.0

^a^MAE: mean absolute error.

**Figure 8:**
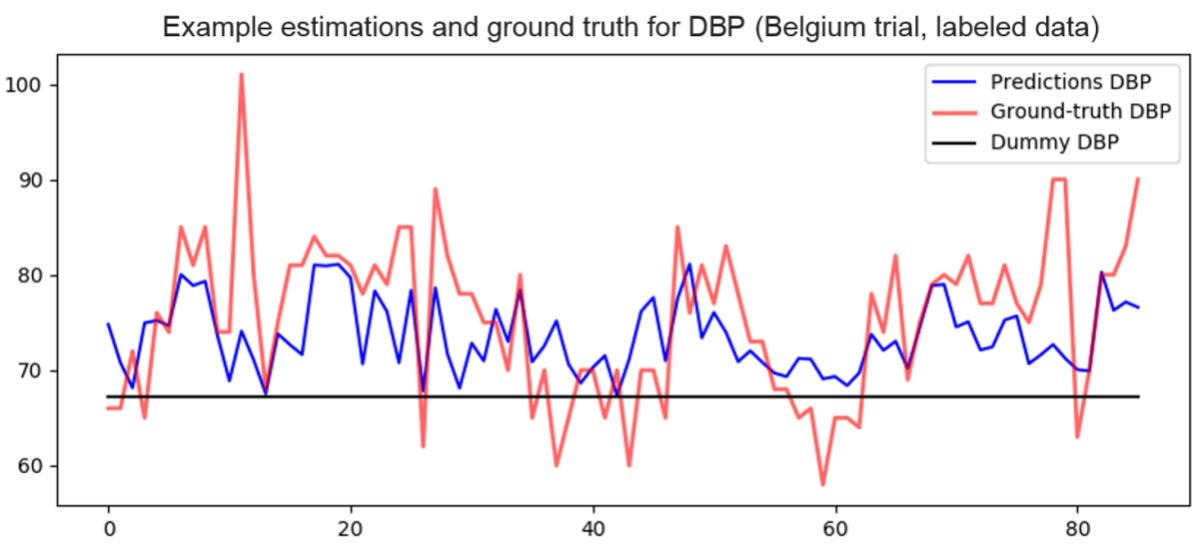
Segment of example estimates and ground truth diastolic blood pressure from the HeartMan trial. DBP: diastolic blood pressure.

An example segment of the DBP estimates is shown in Figure 8. The results show that BP estimation is feasible; however, most state-of-the-art methods are highly dependent on high signal quality to obtain precise morphological features on a per-cycle basis, which is difficult to achieve with an affordable wristband.

#### Psychological Profile Recognition

To test the psychological profile recognition, we collected a data set from 30 healthy subjects (mean age 68, SD 2 years, 6 women and 23 men). The subjects used the HeartMan mobile app for psychophysiological data collection. Leave-one-subject-out evaluation was conducted, and classification accuracy into depressed, anxious, and motivated profiles was used as the evaluation metric. Classification models trained with four machine learning algorithms were compared against a baseline dummy model, which always returned the majority class.

As shown in Table 3, the support vector machine (SVM) model performed best, achieving a fairly high accuracy, especially considering that this is a subject-independent result. In Table 4, we can see the results in terms of precision, recall, and F1-score for the SVM model. The percentages of the confusion matrix as a result of the cross-validation procedure showed that SVM can classify all 3 classes with precisions of 93%, 86%, and 84%, respectively. From the results, it can be observed that the motivated profile was recognized most accurately, whereas most of the misclassifications came from the anxious and depressed profiles, which are sometimes very similar.

**Table 3:** Classification accuracies of the psychological profile detection.

Algorithm	Classification accuracy (%)
Baseline dummy (majority)	37.9
Naïve Bayes	79.7
Multilayer perceptron	75.1
Random forest	62.6
Support vector machine	88.6

**Table 4:** Precision, recall, and F-measures of the psychological profile detection.

Psychological profile	Precision (%)	Recall (%)	F-measure (%)
Motivated profile	93	94	94
Anxious profile	86	83	85
Depressed profile	84	87	86

#### Physical Activity Recognition

The model for physical activity recognition was built and evaluated on recordings of 10 healthy subjects (mean age 59, SD 5 years, 6 women and 4 men). The subjects performed a scenario consisting of all the activities to be recognized with several variations: walking at different speeds, uphill and carrying a burden, eating various foods, and performing a wide range of chores (cooking, sweeping floor, gardening tasks, etc) and hand activities (writing, using a computer, knitting, etc). Similar to the previous cases, a leave-one-subject-out evaluation was conducted. Precision (the fraction of the instances recognized as a certain activity that in fact belong to that activity), recall (the fraction of the instances belonging to a certain activity that are recognized as such), and F-measure (harmonic mean of precision and recall) were used as the evaluation metrics.

Table 5 shows that most of the activities can be recognized reliably. Standing has the smallest F-measure, because it is often misclassified as rest. This is understandable because in both cases, the hand with the wristband does not move much and is not overly problematic because most people rarely stand still for a long time. The second largest problem is confusing eating with hand activities, which is also understandable but makes accurately triggering psychological interventions during eating difficult.

**Table 5:** Precision, recall, and F-measure of the physical activity recognition.

Activity	Precision (%)	Recall (%)	F-measure (%)
Rest	84	89	87
Standing	48	32	38
Walking	75	86	80
Nordic walking	67	78	72
Running	74	62	67
Exercise	72	77	74
Eating	62	61	61
Washing	73	77	75
Chores	84	81	82
Hand activities	67	65	66
Macro average	71	71	71

### General Effectiveness of the System

A proof-of-concept trial was set up to evaluate the effects of the HeartMan intervention on health-related quality of life and disease management (self-care) as primary endpoints [54]. The secondary endpoints we targeted were clinical parameters, illness perception, and mental and sexual health. The clinical trial was registered on NCT03497871 on 2018-04-13. It was implemented in two countries: three hospitals were involved in Belgium, and one hospital and a local health authority participated in Italy. A randomized controlled design was used with a 1:2 ratio of the control and intervention groups. Eligible patients were recruited by the treating cardiologist or general practitioner at the time of regular consultation. After providing informed consent, participants underwent a baseline data collection, containing medical record data registration, questionnaire assessments, and some clinical assessments, including a 6-min walking test. Patients were then randomly assigned to either the control group receiving the usual care or the intervention condition additionally receiving the HeartMan personal health system that they used in their home setting for a period of 3-6 months. All outcome measurements were repeated in both the intervention and control groups at the end of the trial.

The intervention effects were evaluated in a final sample of 56 patients (ie, 34 in the intervention group and 22 in the control group). Trial results showed that the HeartMan system was successful in improving self-care behavior, resulting in a higher quality of disease management, as indicated by the significant (*P*=.02) improvement of 11% in the Self-Care of Heart Failure Index [55]. No such effect was observed on health-related quality of life, as assessed with the Minnesota Living with Heart Failure Questionnaire [56]. Regarding secondary endpoints, using HeartMan significantly (*P*<.001) improved psychological outcomes, that is, intervention patients decreased their level of depression (Beck Depression Inventory II [57]) and anxiety (State Trait Anxiety Inventory Form Y [58]) by 15%, and these reductions were even higher in the patients who had used the mental support module in the app more intensely. The HeartMan intervention also significantly (*P*=.01) reduced the experience of sexual problems, that is, by 26% on the Sexual Adjustment Scale [59]. No effects were shown for illness perception or clinical outcome of exercise capacity. However, additional data available in a subgroup of the trial sample showed a significant (*P*=.04) improvement of 11% in the left ventricular ejection fraction. A more extensive publication of trial results is pending.

### Patients’ Perception of the System

The user experience of HeartMan was investigated both qualitatively and quantitatively in the intervention group. Quantitatively, the Unified Theory of Acceptance and Use of Technology (UTAUT) questionnaire was used [60], adapted to the objectives of the HeartMan system and to the population of older adult users [61]. This questionnaire assesses users’ intentions to use the HeartMan system and their usage behavior. The UTAUT questionnaire pointed out that HeartMan users’ attitude toward the system was generally positive, with low scores on technology anxiety related to this positive attitude and relatively high-performance expectancy (“the degree to which the user expects that using the system will help him or her to attain gains in job performance” [60]).

Qualitatively, semistructured interviews were performed with 10 patients (7 men and 3 women) and their informal caregivers after having participated in the trial for 3-4 months [62]. The results of an in-depth analysis of sociotechnical complexities in home-based health monitoring systems [63] showed some potential for the HeartMan system as a tool for self-management. Although stressful for some participants, collecting health data such as weight and BP in the HeartMan trial generally raised awareness among the patients of their lifestyle and health. Monitoring their health parameters enabled them to be more aware of their bodies, intervene, and ask for help in a timely manner. The evaluations also showed that the HeartMan system positively affected patients’ dietary knowledge and that they felt stimulated to engage in physical activities. This suggests that self-monitoring and empowerment goals are generally achieved. Some weaknesses were also found, such as the need for increased flexibility regarding the interface and interactions with the system.

## Discussion

### Technology

The HeartMan system is complex, spanning sensing devices, a mobile app, and the cloud; combining diverse technologies; and featuring extensive content to comprehensively address CHF management. The challenge of integrating all this was tackled by an architecture with independent components connected through the IoTool middleware as well as the interface and interoperability layer. A lesson learned was that there is a tradeoff between too tight integration, which makes changes difficult, and too many layers between components, which makes integration testing difficult.

Individual components largely performed as expected. BP estimation from PPG proved the most difficult, as this is a difficult research problem even in ideal conditions, when high-quality PPG signals from a clinical or research device are available. Thus, this technology is not yet sufficiently mature for everyday use by patients. In the DSS, we mainly relied on expert knowledge, and only recommendations regarding temperature and humidity were provided by data-based methods. Although we believe data-based decisions will play a greater role in health management in the future, the amount of raw data currently available to support the range of decisions needed to manage a disease such as CHF cannot yet rival the expert knowledge available in the literature and medical practice. Although that knowledge is ultimately based on data, these data are simply not available in one place (and possibly not at all in some cases).

### Medical Perspective

Although the use of telemonitoring systems in cardiac patients has increased tremendously, evidence regarding their effectiveness in managing patients with CHF remains to be mixed [64]. HeartMan, however, is different from most telemonitoring systems: it focuses on empowering patients to properly manage their disease, rather than remote monitoring by health care professionals. It mainly aims to improve the quality of life and self-management in patients by integrating several intervention modalities in the domains of physical health management and psychological support. The trial results showed that the obtained beneficial effects were mostly psychological, more than physical, which is in line with the predefined primary outcomes. A possible explanation is that the system did not achieve sufficient adherence to the advanced and gradually progressive exercise program, which would probably be the most effective way to improve physical health. Nonetheless, before drawing definite conclusions, we need to investigate the effectiveness of the HeartMan system in a wider context, that is, in a larger sample of patients with CHF over a longer intervention period.

### User Perspective

As early as during the analysis of the patients’ context of use, the HeartMan concept was presented to patients and their initial reactions were captured. Several insights gathered in this phase remained relevant during later evaluation phases and applied to patient-monitoring systems in general. One of the most important such insights was the fact that patients tend to have high and not necessarily correct expectations of automatic patient-monitoring systems such as HeartMan. Patients tend to expect their caregivers to be continuously aware of what the system detects. Although this can lead to a positive motivation to monitor health parameters, it can also lead to a false sense of safety. In addition, while many patients were motivated to monitor these health parameters, they were closely related to lifestyle choices, such as nutrition and physical exercise. We learned that several patients disliked the fact that HeartMan monitors these lifestyle choices and are concerned about a possible loss of control and autonomy in this respect.

These observations lead to a nuanced view of the patients’ perspective on self-monitoring technology, with both perceived benefits (feeling of reassurance, increased awareness) and drawbacks (false perception of safety and loss of autonomy). This view suggests that patient empowerment truly is the correct goal, in the sense that patients should not rely on the supervision of caregivers (as it may not be available) and should also not feel judged and controlled by the system (but should be making healthy lifestyle choices for themselves). We also observe that although HeartMan started on the way to this goal, further improvements can still be made.

On a more practical level, we learned that a distinction between patients regarding digital literacy can be useful [29]. The patients with high literacy received a full explanation of HeartMan functionality at the beginning of the trial. They were encouraged to be proactive and to navigate through the various functions of the application, which was empowering. Such use was feasible because the interface, particularly the information hierarchy of the application, was designed, tested, and refined in collaboration with the patients. Patients with lower digital literacy were asked to react primarily to notifications in the app. In this way, they were able to cope with the app that, even though it was designed to be simple, it was still relatively complex for some users.

### Conclusions

We developed HeartMan, a personal health system for the comprehensive self-management of CHF. It uses a wristband and other sensing devices to obtain information on the patient’s BP, physical activity, and psychological profile by means of machine learning as well as some other parameters by more mundane means. All this information is fed into a DSS, which provides recommendations on physical health and psychological support. These translate into a detailed physical exercise program, mindfulness exercises, games, and other forms of support for the patient. This is adapted to the patient’s physical capacity, current activity, and psychological profile. A web application for medical professionals is also a part of the system. Patients with CHF were involved throughout the development of the system to ensure the system meets their needs. The final prototype was evaluated in a proof-of-concept trial in 56 patients, showing significantly improved disease management while reducing depression, anxiety, and sexual problems. Although illness perception and exercise capacity did not improve, a significant improvement in left ventricular ejection fraction was observed in a subgroup. Overall, the patients’ perception of the system was positive.

The HeartMan system was designed with both patients and medical professionals. It works best when integrated with a hospital information system to have access to the users’ up-to-date health records and to provide information on the users to their treating clinicians. As such, it bridges the gap between user-friendly mHealth solutions and medical devices, but it can only be offered to patients through a health provider. Therefore, we are also working on a simplified version of the system that will not be a medical device from a regulatory perspective and will not require connection to a hospital or any kind of backend. This will make it easily deployable via mobile app stores and widely accessible to patients with CHF.

In summary, the HeartMan project combined a range of advanced technologies with human-centered design to develop a complex system that was shown to help patients with CHF. Its benefits were psychological more than physical, which may be because the system did not manage to cause difficult behavioral changes such as increased exercise. The reason for this may be that the system was designed to be more supportive than persuasive. Thus, a key area for future development should be behavior change techniques. Nevertheless, the system is ready to be used, and we are pursuing multiple paths to the market.

## Abbreviations

BP: blood pressure
CHF: congestive heart failure
DBP: diastolic blood pressure
DSS: decision support system
FHIR: fast health care interoperability resource
MAE: mean absolute error
mHealth: mobile health
PPG: photoplethysmogram
SBP: systolic blood pressure
SVM: support vector machine
UTAUT: Unified Theory of Acceptance and Use of Technology
